# Community characteristics of the gut microbiomes of competitive cyclists

**DOI:** 10.1186/s40168-017-0320-4

**Published:** 2017-08-10

**Authors:** Lauren M. Petersen, Eddy J. Bautista, Hoan Nguyen, Blake M. Hanson, Lei Chen, Sai H. Lek, Erica Sodergren, George M. Weinstock

**Affiliations:** 1 The Jackson Laboratory for Genomic Medicine, 10 Discovery Drive, Farmington, CT 06032 USA; 2 Corporación Colombiana de Investigación Agropecuaria, Km 14 Vía Mosquera-Bogotá, Mosquera, Cundinamarca Colombia

**Keywords:** Gut microbiome, Athletes, Cyclists, Exercise, Microbiota, Metagenomics, Metatranscriptomics

## Abstract

**Background:**

Changes in diet and exercise can alter the gut microbiome of humans and mice; however, few studies to date have assessed the microbiomes of highly fit athletes. In this pilot study, we used metagenomic whole genome shotgun (mWGS) and metatranscriptomic (RNA-Seq) sequencing to show what organisms are both present and active in the gut microbiomes of both professional and amateur level competitive cyclists and to determine if any significant differences exist between these two groups.

**Results:**

Using mWGS sequencing data, we showed that the gut microbiomes of 33 cyclists split into three taxonomic clusters, characterized by either high *Prevotella*, high *Bacteroides* or a mix of many genera including *Bacteroides*, *Prevotella*, *Eubacterium*, *Ruminococcus*, and *Akkermansia*. While no significant correlations could be found between taxonomic cluster and being either a professional or amateur level cyclist, high abundance of the genus *Prevotella* (≥2.5%) was significantly correlated with time reported exercising during an average week. Increased abundance of *Prevotella* was correlated with a number of amino acid and carbohydrate metabolism pathways, including branched chain amino acid metabolism. Further analysis of the metatranscriptome revealed significant taxonomic differences when compared to the metagenome. There was increased abundance of *Methanobrevibacter smithii* transcripts in a number of professional cyclists in comparison to amateur cyclists and this archaeon had upregulation of genes involved in the production of methane. Furthermore, when methane metabolism was upregulated, there was similar upregulation of energy and carbohydrate metabolism pathways.

**Conclusions:**

These results provide a framework for common constituents of the gut community in individuals who follow an exercise-rich lifestyle. These data also suggest how certain organisms such as *M. smithii* may beneficially influence the metabolic efficiency of the gut community in professional cyclists due to synergistic metabolic cross-feeding events.

**Electronic supplementary material:**

The online version of this article (doi:10.1186/s40168-017-0320-4) contains supplementary material, which is available to authorized users.

## Background

The human gut microbiome is essential to health, playing a vital role in host physiology, metabolism, nutrition, and immune system development. Studies such as the Human Microbiome Project (HMP) and MetaHIT showed that the gut microbiome is comprised of thousands of different bacterial taxa as well as various archaea, eukaryotic microbes, and viruses [1, 2]. Factors such as lifestyle, age, genetics, and diet can change the gut microbiome, resulting in an ecosystem that is highly dynamic [3–6]. However, few studies have focused on what impact exercise has on the gut microbiome and, to date, all but two of these studies utilized murine models [7, 8]. Still, these preliminary studies suggest exercise influences the composition of the gut microbial community.

A common finding in murine and human studies looking at the effects of exercise on the gut microbiome is an increase in species richness (alpha diversity) [7]. Evans et al. used a mouse model to demonstrate that a combination of exercise and diet has a stronger influence on the composition of the gut microbiota than diet alone, with greater alpha diversity in exercised mice vs. sedentary mice even when fed the same diet [9]. Several other studies using murine-based models also demonstrated increased alpha diversity in animals that exercised vs. those that were sedentary [10–12]. However, there was little agreement in what taxa were influenced by exercise. Other than a positive correlation between exercise and *Lactobacillus* [10–12], there are no other taxa that consistently increase in relative abundance in exercised mice or rats.

The studies published to date on human subjects provide only a first glimpse into how exercise can influence the human gut microbiome. Clarke et al. showed that Irish rugby players had increased microbial diversity compared to a healthy control cohort, though it was unclear if this effect was due to exercise, a high protein diet, or a combination of the two factors [13]. Rugby players had lower overall abundances of *Bacteroides* and *Lactobacillus* and enrichment for *Akkermansia muciniphila*, a mucin-degrading microbe that is negatively correlated with obesity and metabolic syndrome [14]. mWGS analysis further revealed that the gut microbiomes of these rugby players were enriched for pathways involved in amino acid biosynthesis, carbohydrate metabolism, and short-chain fatty acid (SCFA) synthesis [15]. Estaki et al. investigated the link between cardiorespiratory fitness and the gut microbiome in human subjects and found peak oxygen uptake to correlate with alpha diversity [16]. This diversity correlated with certain microbial metabolic functions including chemotaxis, motility, and fatty acid biosynthesis. High cardiorespiratory fitness also correlated with an increase in the SCFA butyrate, a finding that agrees with a murine study that also showed an increase in fecal butyrate concentration in exercised rats [17]. Increases in fecal butyrate were found when relative abundances of *Clostridiales*, *Roseburia*, *Lachnospiraceae*, and *Erysipelotrichaceae* were increased [16]. The health-related effects of butyrate have been investigated, and the benefits of this SCFA are numerous. Butyrate has anti-carcinogenic and anti-inflammatory properties, directly feeds colonocytes, and can also affect satiety [18]. Butyrate, along with propionate and acetate, also provides ~10% of the daily caloric requirements in humans [18].

Understanding whether microbes play a pivotal role in athletic performance is of particular interest to athletes who work to improve their results in competition as well as reduce recovery time during training. Moreover, such knowledge may be of general benefit to human health. To accomplish this, further studies are required to understand how the microbiome influences athletes’ success in competition by way of anti-inflammatory effects, optimal breakdown and utilization of consumed food, and other beneficial effects for overall health. To extend our definition of the ‘healthy microbiome,’ as well as to investigate how exercise influences the gut community, we conducted a pilot project to study the gut (stool) microbiomes of 22 professional and 11 amateur competitive cyclists. Cycling is a sport that requires high cardiorespiratory fitness, strength, and upwards of 20–30 h/week of training to compete at the elite level. Therefore, we sought to determine if there were differences in the gut microbiomes between professional and amateur level cyclists. Here, we report metagenomic whole genome shotgun sequencing (mWGS) and RNA sequencing (RNA-Seq) analyses that characterize each cyclist’s microbial community, identify genetic capabilities of those communities, measure gene expression patterns, and identify potential characteristics associated with extraordinary fitness.

## Results

### Taxonomic clustering identified three microbial communities by mWGS sequencing

**Fig. 1:**
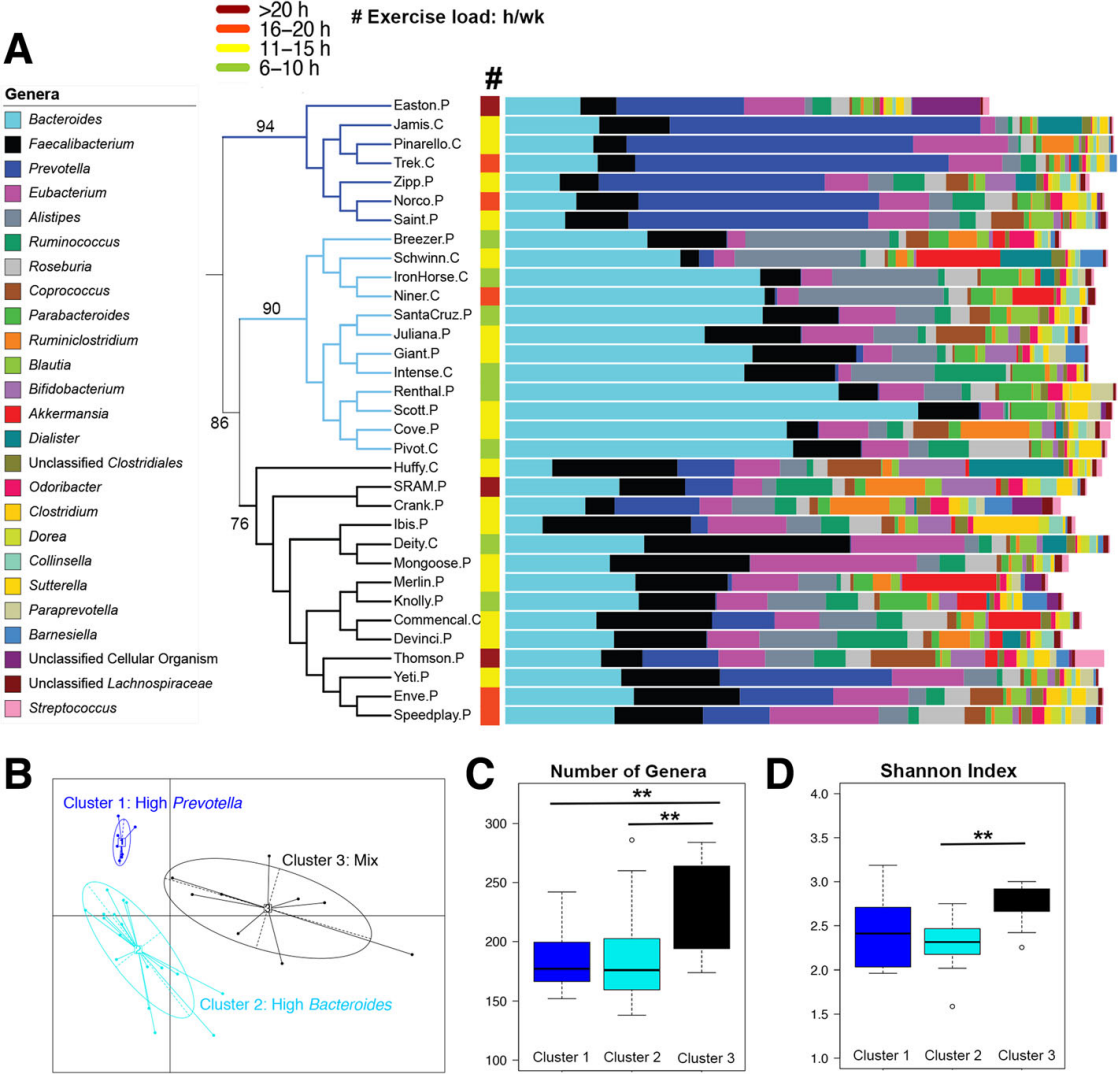
Identification of three taxonomic clusters in cyclists. **a** Dendrogram using the BC dissimilarity index of the top 25 genus-level relative abundance profiles as determined with mWGS sequencing. Genera in the dataset are listed in the key on the left in descending order, with *Bacteroides* on top as the most abundant organism across all the samples. Included on the dendrogram branches are assigned AU *p* values. For dendrogram labels, ‘*C*’ indicates a CAT 1 (amateur) cyclist and ‘*P*’ depicts a professional cyclist. The *color strip* marked with the pound sign (#) indicates the average amount of exercise completed each week. **b** The three principal coordinates of the Jensen-Shannon distances generated from the cyclists’ genus-level relative abundance profiles. Samples are colored by cluster as identified by the partitioning around medoids (PAM) clustering algorithm. *Dark blue* is cluster one, *light blue* is cluster two, and *black* is cluster three. **c** Cyclists in cluster three overall had a higher number of genera (higher richness) than those in cluster one (*p* = 0.0112) and cluster two (*p* = 0.0154). **d** The Shannon diversity index was significantly higher for cluster three compared to cluster two (*p* = 0.0004) but was not significantly different than cluster one (*p* = 0.0534). Statistical significance was determined by the Wilcoxon rank-sum test for each pairwise comparison

Fecal DNA from 33 cyclists was sequenced by mWGS sequencing and resulting reads were mapped to the Real Time Genomics (RTG) database (see Additional file 1 for sequencing depth). Relative abundance tables were generated (Additional file 2) and the top 25 most abundant genera across all 33 samples was determined and plotted in a dendrogram using the Bray-Curtis (BC) distance measurement for clustering (Fig. 1a). To assess confidence in the clustering, approximately unbiased (AU) *p* values were generated and the strongest cluster (AU *p* value of 94) within the dendrogram, cluster 1, was characterized by a high relative abundance of the genus *Prevotella* and low abundances of *Bacteroides*. A second cluster of 12 cyclists (AU *p* value of 90) was characterized by a high relative abundance of *Bacteroides* and either low or no *Prevotella*. The third cluster (AU *p* value of 76), containing the remaining 14 cyclists, did not have a single genera driving the community but instead was characterized by a mix of genera including *Bacteroides*, *Prevotella*, *Eubacterium*, *Ruminococcus*, and *Akkermansia*. We applied the partitioning around medoids (PAM) method using Jensen-Shannon (JS) distance [19] to confirm the presence of three clusters (Fig. 1b). A separate analysis using 16S rRNA gene sequencing using the same methods supported the presence of three clusters (Additional file 3).

To determine what factors correlated with each mWGS taxonomic cluster, we first measured richness and alpha diversity. Cyclists in cluster three had a greater number of genera in their gut communities than those cyclists in cluster one (*p* = 0.0112) and cluster two (*p* = 0.0154, Fig. 1c). Shannon diversity index scores showed that cluster three was significantly more diverse than cluster two (*p* = 0.0004) but was not significantly different than cluster one (*p* = 0.0534). We then looked for correlations using Spearman’s rank (ρ) between each cluster and factors reported in the metadata questionnaires (Table 1). These factors included the amount of time spent exercising per week, gender, and overall diet. However, no significant correlations were identified. Additionally, no significant correlation was found between race category (i.e., professional or amateur) and taxonomic cluster; however, there were a greater number of professional cyclists than amateur cyclists in cluster three (11 professionals vs. 3 amateurs).

**Table 1:** Reported metadata (diet, alcohol consumption, exercise load), *Prevotella* abundance, mWGS taxonomic cluster, and race category (as recorded from usacycling.com)

Cyclist	Sex	Diet	# alcohol beverages per week	Exercise load (h/week)	% abundance Prevotella (mWGS)	Taxonomic cluster (mWGS)	Race category
Knolly	F	Equal protein, fat, carbs	1–5	6–10	0.20%	3	PRO
Santa Cruz	F	Equal protein, fat, carbs	0	6–10	0.17%	2	PRO
Pivot	M	Equal protein, fat, carbs	0	6–10	0.13%	2	CAT 1
Breezer	F	Equal protein, fat, carbs	6–10	6–10	0.13%	2	PRO
Intense	F	Vegetarian	1–5	6–10	0.49%	2	CAT 1
Deity	M	Equal protein, fat, carbs	1–5	6–10	0.13%	3	CAT 1
Renthal	M	Equal protein, fat, carbs	1–5	6–10	0.20%	2	PRO
Iron Horse	M	Equal protein, fat, carbs	1–5	6–10	0.13%	2	CAT 1
Scott	M	Equal protein, fat, carbs	1–5	11–15	0.33%	2	PRO
Devinci	M	Equal protein, fat, carbs	1–5	11–15	0.15%	3	PRO
Ibis	M	Equal protein, fat, carbs	1–5	11–15	2.65%	3	PRO
Juliana	M	Equal protein, fat, carbs	1–5	11–15	0.18%	2	PRO
Merlin	M	High complex carbs	1–5	11–15	0.70%	3	PRO
Schwinn	M	Paleo	0	11–15	2.35%	2	CAT 1
Mongoose	M	Equal protein, fat, carbs	1–5	11–15	0.08%	3	PRO
Huffy	F	Paleo	0	11–15	9.02%	3	CAT 1
Giant	M	Equal protein, fat, carbs	0	11–15	1.12%	2	PRO
Commencal	M	Paleo	1–5	11–15	9.93%	3	CAT 1
Cove	F	Paleo	1–5	11–15	0.19%	2	PRO
Jamis	M	Equal protein, fat, carbs	15+	11–15	49.11%	1	CAT 1
Yeti	F	Gluten-free	1–5	11–15	27.18%	3	PRO
Zipp	M	Equal protein, fat, carbs	1–5	11–15	35.66%	1	PRO
Saint	F	Equal protein, fat, carbs	0	11–15	38.19%	1	PRO
Crank	F	Paleo	0	11–15	14.67%	1	PRO
Pinarello	M	Equal protein, fat, carbs	0	11–15	45.27%	1	CAT 1
Trek	M	Equal protein, fat, carbs	1–5	16–20	49.52%	1	CAT 1
Niner	F	Paleo	1–5	16–20	0.36%	2	CAT 1
Norco	M	High complex carbs	6–10	16–20	38.47%	1	PRO
Enve	M	Equal protein, fat, carbs	1–5	16–20	14.74%	3	PRO
SpeedPlay	M	Equal protein, fat, carbs	1–5	16–20	10.53%	3	PRO
SRAM	M	Equal protein, fat, carbs	1–5	20+	7.53%	3	PRO
Easton	M	High complex carbs	1–5	20+	27.03%	1	PRO
Thomson	F	Gluten-free	0	20+	12.12%	3	PRO

### Amount of exercise correlates with greater *Prevotella* abundance

**Fig. 2:**
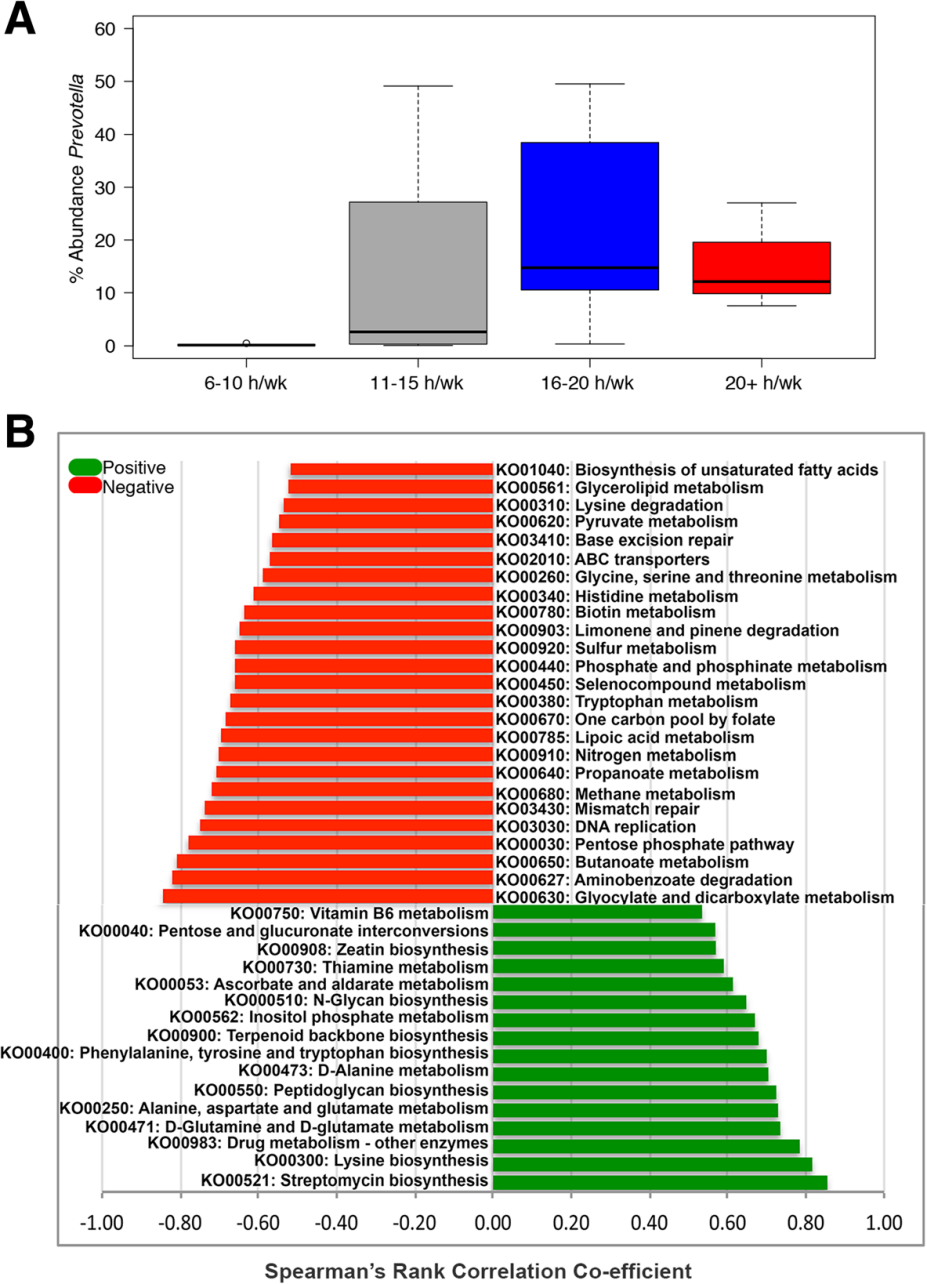
*Prevotella* abundance is significantly correlated to exercise load and a number of KEGG pathways. **a** Box plot showing the average abundance of *Prevotella* in the gut microbiomes of cyclists who reported either 6–10, 11–15, 16–20, or 20+ hours of exercise per week. Fisher’s exact test was used to determine that cyclists who exercised >11 h/week were more likely to have ≥2.5% *Prevotella* (*p =* 0.0026). **b** Histogram showing significant positive (*green*) or negative (*red*) correlations between abundance of *Prevotella* and abundance of KEGG pathways. Correlations were calculated using Spearman’s rank (*p* < 0.05)

Because no correlations were found between any factors reported in the metadata and communities defined by taxonomic cluster, we looked for correlations between metadata and abundances of single genera. From these analyses, we found a significant correlation between exercise load and the abundance of *Prevotella*. The abundance of *Prevotella* was highest for cyclists reporting either 20+ or 16–20 h of exercise per week, with median abundances of 14.75 and 12.12%, respectively (Fig. 2a). The eight cyclists that reported exercising 6–10 h/week had a median abundance of only 0.15% *Prevotella*. Overall, Fisher’s exact test showed that cyclists who exercised >11 h/week were more likely to have ≥2.5% *Prevotella* (*p* = 0.0026, Table 1). This finding was independent of whether the cyclist was a professional or amateur level racer.

### Associations between *Prevotella* abundance and metabolic function

For metabolic function assignment, mWGS reads were assessed for functional activity by alignment to the KEGG database and corresponding relative abundance profiles were generated for all results (Additional file 4). Correlations between KEGG pathways and the abundance of *Prevotella* were analyzed. *Prevotella* was positively correlated (ρ > 0.5, *p* < 0.01) to a number of amino acid metabolism pathways, including lysine biosynthesis, alanine, aspartate and glutamate metabolism, and D-glutamine and D-glutamate metabolism (Fig. 2b). *Prevotella* was also significantly correlated to multiple pathways involved in drug metabolism, carbohydrate metabolism, and metabolism of cofactors and vitamins, including vitamin B6 metabolism. *Prevotella* was negatively correlated with a number of other pathways involved in amino acid metabolism and carbohydrate metabolism, including short chain fatty acid (SCFA) metabolism as well as pathways involving nitrogen, sulfur, and methane metabolism.

We evaluated the species of *Prevotella* within samples that contained ≥2.5% *Prevotella* to determine if there was one species driving these correlations with metabolic function. This showed that *Prevotella copri* was the most abundant species (51–98% of the total *Prevotella* community) in every sample (Additional files 5 and 6A). Two cyclists, Enve and Easton, showed high relative abundance of *Prevotella stercorea* as well (40.17 and 34.35%, respectively). This was in contrast to the number of *Bacteroides* species found in each of the 33 cyclists (Additional file 6B). All 33 cyclist samples were characterized by a wide variety of *Bacteroides* species. These differences, however, could be attributed to the number of fully sequenced genomes; at time of publication, only two genomes of both *P. copri* and *P. stercorea* were available, and all four were incomplete, while there were dozens of sequenced *Bacteroides* genomes.

A separate analysis based on identifying operational taxonomic units (OTUs) with 16S rRNA sequencing reads identified 11 different *Prevotella* OTUs in the 17 cyclists with ≥2.5% *Prevotella.* Five of the 11 OTUs mapped to *P. copri* (OTU1, OTU10, OTU380, OTU515, and OTU573), however, with varying percent identities (93–99%), indicating a likelihood of several strains of *P. copri* and possibly different species for OTUs with identities of <97%. The remaining *Prevotella* OTUs had closest hits to other *Prevotella* species. To determine which OTUs were most dominant in cyclists with ≥2.5% *Prevotella*, the total community of *Prevotella* in each sample was normalized to 100% and relative abundances of *Prevotella* OTUs were calculated (Additional file 7). In agreement with mWGS abundance data, *P. copri* (OTU1) was the dominant species in the cyclists based on 16S rRNA reads with the exception of cyclist Giant, whose sample had <2.5% *Prevotella* based on mWGS data.

### Metatranscriptomic sequencing reveals further insight into the gut microbiomes of cyclists

For further characterization of the gut microbiomes of cyclists and to gain further insight into the most active taxa and their metabolism, metatranscriptomic sequencing (RNA-Seq) was performed on all 33 cyclists’ fecal samples. Alignment of RNA-Seq reads to the RTG database revealed differences in taxonomic composition when compared to mWGS. At the phylum level, *Bacteroidetes* was highly abundant from mWGS analysis, but RNA-Seq showed that overall mRNA relative abundance of *Bacteroidetes* organisms was significantly lower than DNA abundance (*p* < 0.001, Additional files 8, 9, and 10). Conversely, *Firmicutes* had greater abundance in the metatranscriptome than the metagenome, indicating *Firmicutes* were more active in the gut than *Bacteroidetes* (*p* < 0.001). *Euryarchaeota* also had higher abundance of mRNA transcripts vs. DNA reads, indicating highly active archaea in the gut in a number of professional cyclists (*p* < 0.001).

**Fig. 3:**
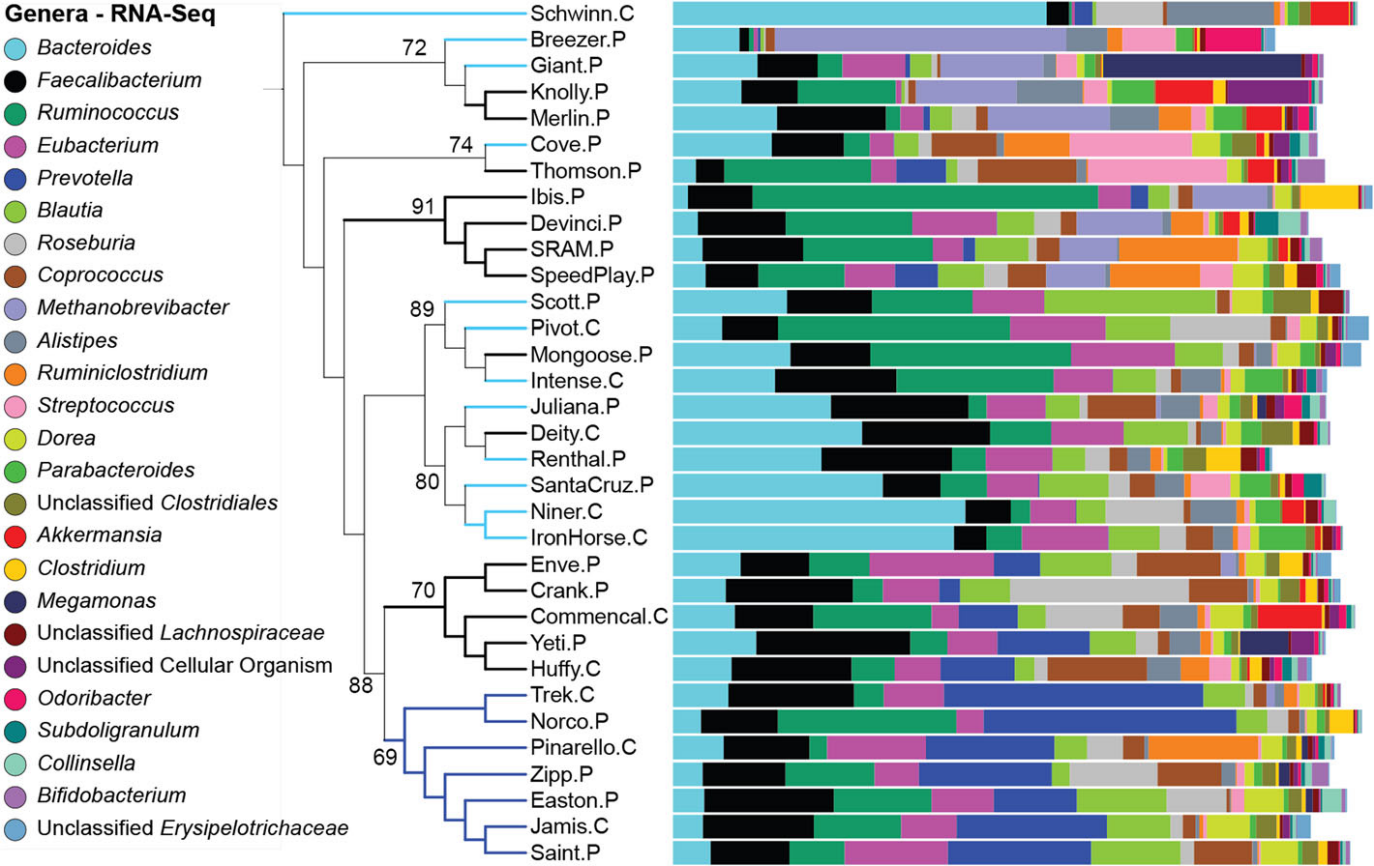
Taxonomic composition of the metatranscriptome. Dendrogram of the hierarchical clustering of relative abundance profiles for the top 25 genera as measured with mRNA transcripts for all 33 cyclists. Genera in the dataset are listed in the key on the left in descending order, with *Bacteroides* on top as the most abundant organism across all the samples. Included on the dendrogram branches are assigned AU *p* values. Clustering was performed using the BC distance metric and average-linkage method. The colors of the branches reflect the mWGS cluster that cyclist was in as shown in Fig. 1 (*dark blue* is cluster 1, *light blue* cluster 2, and *black* cluster 3). For cyclist sample names, ‘*C*’ indicates a CAT 1 (amateur) cyclist and ‘*P*’ depicts a professional cyclist

The differences between the metagenomic and metatranscriptomic taxonomic composition was also seen at the genus level (see Additional file 11 for genus-level abundances). While *Bacteroides*, *Faecalibacterium*, and *Eubacterium* still ranked as first, second, and fourth most abundant genera overall with RNA-Seq, *Ruminococcus* went from the sixth most abundant genera to third when measuring mRNA (Fig. 3). In addition, while *Prevotella* was the third most abundant genera in mWGS sequencing, it was the fifth most abundant genus based on mRNA transcripts. The seven cyclists that formed cluster one when analyzing mWGS taxonomic data (see Fig. 1a) still clustered together, however, with less significance (AU *p* value of 69). The previously defined clusters 2 and 3 did not exist in the metatranscriptome as cyclists from those two groups mixed together into smaller, often less statistically significant clusters.

**Fig. 4:**
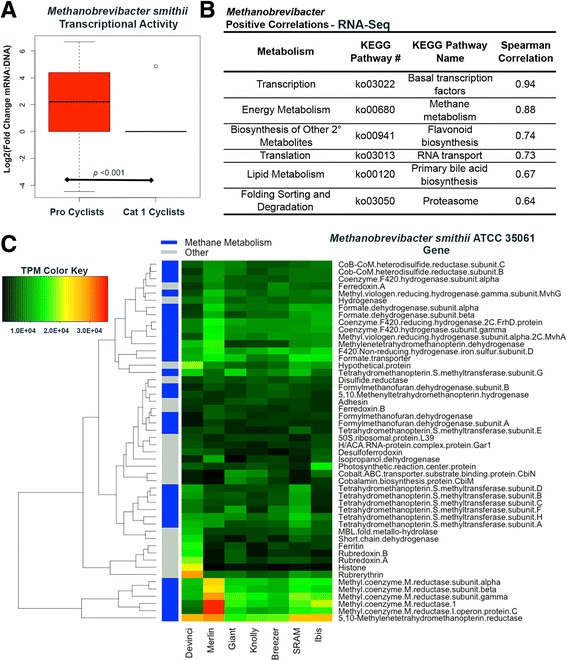
Characterization of *Methanobrevibacter smithii* transcriptional activity. **a** A box plot demonstrating the ratio of mRNA abundance to DNA abundance showed higher transcriptional activity by *M. smithii* in professional cyclists vs. CAT 1 cyclists (***p < 0.01). **b** This increased activity by *M. smithii* directly correlated to six upregulated KEGG pathways, including methane metabolism (*p* < 0.001). **c** RNA-Seq reads from seven professional cyclists with high *M. smithii* activity were mapped to the genome of reference strain *M. smithii* ATCC 35061. The top 50 most highly expressed genes, as determined by TPM, are presented in a heat map with clustering of genes determined using the BC distance metric. The *color strip* indicates whether the gene is involved in methane metabolism (*blue*) or is involved in a separate KEGG pathway (*gray*)

Perhaps the most striking difference between the metagenome and metatranscriptome was the significant increase in mRNA reads (compared to DNA reads) mapping to *Methanobrevibacter* in a number of professional cyclists. The species abundance tables (Additional file 12) showed this was due to transcriptional activity of *Methanobrevibacter smithii*. *M. smithii* gene expression was highly variable between cyclists but was highest in professional-level cyclists compared to CAT 1 cyclists as determined with Fisher’s exact test (*p* < 0.001, see Additional file 13 for summary of *M. smithii* abundance in all cyclists). Transcriptional activity by *M. smithii* was identified in 15/22 professional cyclists compared to only 1/11 CAT 1 cyclists with relative abundances ranging from 0.2 to 41.0% in the professional cyclists. The ratio between *M. smithii* mRNA vs. DNA relative abundance was as high as 102-fold. The fold changes were log2 transformed and plotted to show the significant difference between professional cyclists and CAT 1 cyclists (*p* < 0.01, Fig. 4a). Because some samples showed the presence of *M. smithii* in RNA-Seq analysis but not in mWGS data, qPCR was done on all mWGS samples using 16S primers designed for *M. smithii.* The results confirmed the presence of this archaeon in fecal gDNA from cyclists that had *M. smithii* in RNA-Seq analysis with the exception of cyclists Yeti (Additional file 13).

### Influence of transcriptional activity by *Prevotella* and *Methanobrevibacter* on metabolic functions in the gut community

For insight into what metabolic functions correlate with transcriptional activity by *Prevotella* and *Methanobrevibacter*, Spearman’s rank correlation coefficients (ρ) were determined between these two genera and KEGG pathway analyses performed with RNA-Seq reads (see Additional file 14 for all KEGG pathway abundances). *Prevotella* transcriptional activity was positively correlated to only three KEGG pathways; drug metabolism, valine, leucine, and isoleucine biosynthesis, and D-glutamine and D-glutamate metabolism. *Prevotella* transcriptional activity was negatively correlated to two amino acid metabolism pathways; lysine degradation and tryptophan metabolism.

*Methanobrevibacter* spp*.* are methane producers, and our Spearman’s rank correlation data shows positive associations between *Methanobrevibacter* and methane metabolism (ρ = 0.88, *p* < 0.01) (Fig. 4b). Other significant positive correlations (ρ ≥ 0.5, *p* < 0.01) include pathways involved in transcription and translation, secondary metabolite synthesis, and lipid metabolism. There was only one significant negative correlation found with RNA-Seq data and that was streptomycin biosynthesis, a pathway positively correlated with *Prevotella* abundance.

### Further insight into *Methanobrevibacter smithii* and methane metabolism

To gain further insight into the transcriptional activity of *M. smithii*, with the goal of uncovering why high metabolic activity by this archaeon would benefit professional cyclists, RNA-Seq reads from eight of the professional cyclists with high *M. smithii* mRNA abundance (≥8.0%) were aligned to the reference *M. smithii* ATCC 35061. One set of RNA-Seq reads, from cyclist Speedplay, showed poor alignment to the reference compared to the seven other samples (which all showed strong coverage across the entire genome) and therefore was discarded from further analysis. The top 50 most expressed genes across all remaining seven samples (professional cyclists Devinci, Merlin, Giant, Knolly, Breezer, SRAM, and Ibis) were plotted and genes with similar expression profiles were clustered using the BC dissimilarity index (Fig. 4c). Thirty of the 50 most expressed genes were involved in methane metabolism (KEGG pathway ko00680). Those genes with highest expression (1.2E + 04–4.0E + 04 transcripts per million (TPM)) were the subunits coding for methyl-coenzyme M reductase. Methyl-coenzyme M reductase catalyzes the reduction of methyl-coenzyme M and coenzyme B to methane, which is the final step in methane biosynthesis. Another highly expressed cluster was those genes responsible for formate dehydrogenase, which is an enzyme that catalyzes the reaction that oxidizes formate to produce reduced coenzyme F420. A third cluster of highly expressed genes important for methane metabolism included genes the subunits of tetrahydromethanopterin S-methanyltransferase, an enzyme important in the synthesis of methane from CO_2_. Other highly expressed genes not involved in methane metabolism included those involved in oxidative stress tolerance, such as rubrerythrin and several rubredoxin genes.

**Fig. 5:**
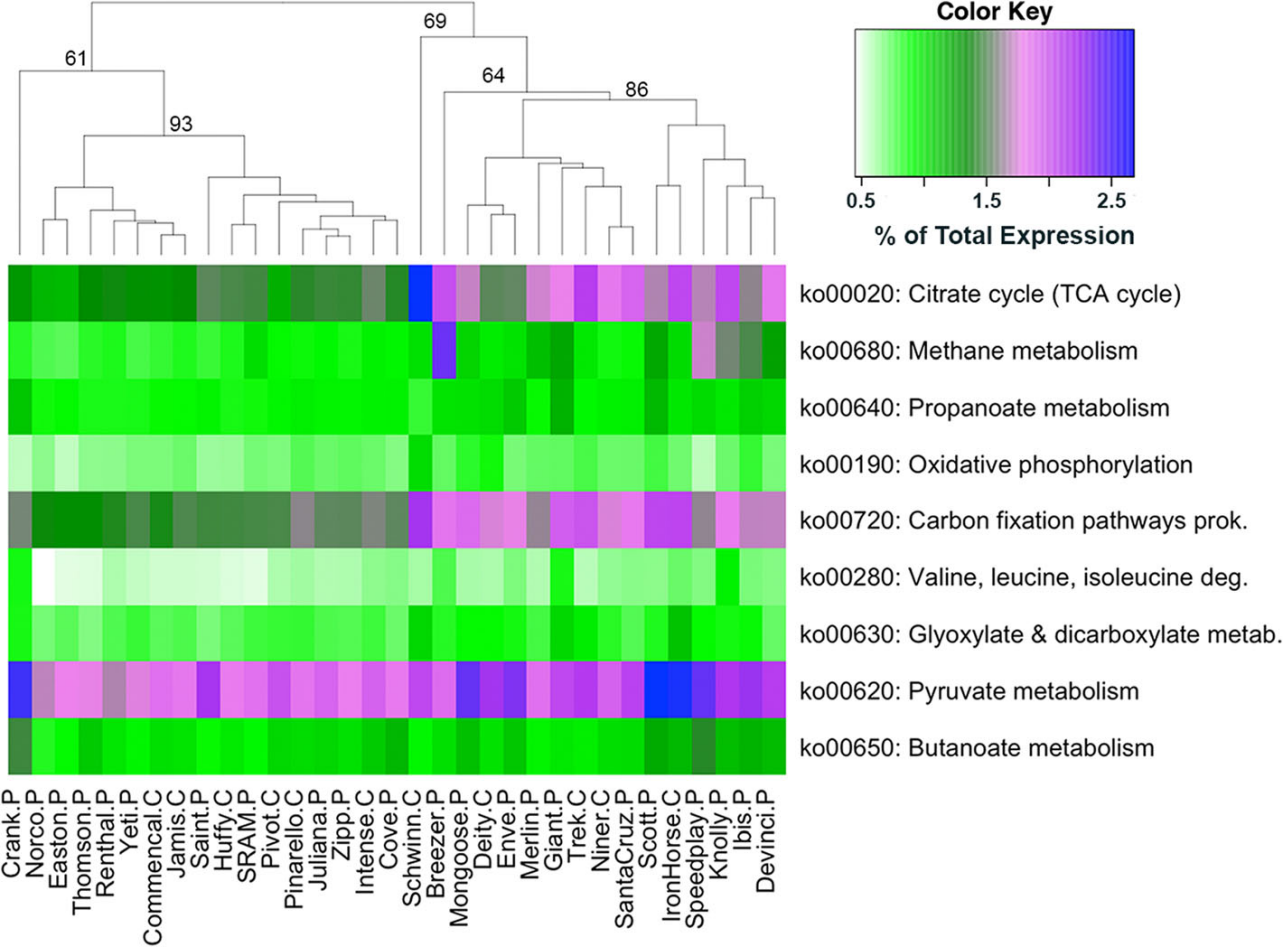
Metabolic pathways important for carbohydrate metabolism and energy production were upregulated in conjunction with methane metabolism. Spearman’s rank correlation coefficients and corresponding *p* values were calculated in R to determine what KEGG pathways were upregulated along with methane metabolism. A heatmap using expression data of these pathways was generated with cyclists’ samples clustered using the BC distance metric. Included on the dendrogram branches are assigned AU *p* values. All nine pathways shown are significantly correlated with each other (*p* < 0.05). For cyclist sample names, ‘*C*’ indicates a CAT 1 (amateur) cyclist and ‘*P*’ depicts a professional cyclist

To further assess the impact of high methane metabolism activity on the community as a whole, correlations between methane metabolism and all other KEGG pathways were investigated using RNA-Seq data. We found that when methane metabolism was upregulated, five carbohydrate metabolism and three energy metabolism were similarly upregulated (ρ ≥ 0.4, *p* < 0.01) (Fig. 5). These pathways include the citrate cycle, oxidative phosphorylation, and pyruvate metabolism. Pathways in SCFA production, propanoate metabolism and butanoate metabolism, were similarly upregulated along with methane metabolism. Using the Bray-Curtis (BC) dissimilarity index and the average-linkage method, cyclists split into two major clusters (with outliers Crank, Breezer, and Schwinn) based on these nine KEGG pathways. The separation into these two clusters was primarily due to the activity of the citrate cycle, carbon fixation, and methane metabolism. There was no correlation between these two clusters and the three taxonomic clusters based on mWGS sequence data.

## Discussion

This pilot study provides one of the first in-depth investigations of the gut microbiomes of athletes and highlights the diversity of microorganisms at the metagenomic and metatranscriptomic level. However, because this was an exploratory study, we acknowledge the limitations of the data presented here including lack of in-depth dietary analysis and a matching non-cyclist cohort. For future studies, we will be recruiting more cyclists, will be including a healthy non-athlete cohort, and will be including diet and exercise information as it is likely diet is playing a role alongside exercise in influencing the taxonomic composition of the cyclists’ gut microbiomes [20–24]. Nevertheless, the data presented here provides valuable insight into the gut communities of cyclists.

In agreement with Clarke et al. [13], we found low abundances of *Bacteroides* in our athletes. In addition, 30 out of 33 cyclists in this study had *Akkermansia*, with seven cyclists having relative abundances of >2% of this microbe in their metagenomic community. In addition to these observations, this study has uncovered several other important distinctions about the microbiomes of cyclists that, to our knowledge, have not been described previously. First was the high relative abundance of *Prevotella* in cyclists that spend >11 h/week training. Despite having been defined as a driver of one of the previously defined enterotypes [19], *Prevotella* is normally found in only a small percentage of healthy individuals in European and American cohorts [1, 4, 25, 26]*.* Previous microbiome studies have repeatedly identified correlations of both diet and geographic location to abundances of *Prevotella* or *Bacteroides. Prevotella* is more often found in individuals from certain areas of Asia [27, 28] and rural Africa [29], and this enrichment for *Prevotella* is often reflective of diets high in complex carbohydrates (including high dietary fiber from various sources including fruits and vegetables), egg food items, and high vitamins and minerals [28, 30]. *Prevotella* abundance has also been correlated to the number of average kilocalories consumed per day [31]. Although the participants in the Noguera-Julian et al. study did not report on exercise [31], it is possible they lived active lifestyles given that they consumed more kilocalories but had the same average body mass index as other participants in the study who consumed less kilocalories. Endurance athletes, especially cyclists, are well known to have diets high in kilocalories, comprised of both simple and complex carbohydrates [32, 33] and to supplement with various kinds of over-the-counter vitamins [34]. Though we did not conduct in-depth questionnaires on diet, these cyclists do consume a high amount of carbohydrates for training and during races. Therefore, we hypothesize that these cyclists likely have high *Prevotella* due to, at least in part, a diet high in carbohydrates, high caloric intake, and a substantial number of hours spent exercising on a weekly basis. Furthermore, we hypothesize that cyclists have different species of *Prevotella* that have yet to be characterized as our 16S OTU analysis identified 11 different *Prevotella* OTUs across all 33 cyclists but only 4 of these OTUs were ≥97% identical to the closest taxonomic hit, and none had 100% identity.

Metatranscriptome analysis uncovered further characteristics of the cyclists’ microbial communities. In agreement with a previous study utilizing both mWGS and RNA-Seq platforms to characterize the gut microbiome [35], the taxonomic metatranscriptomic profiles for many of the cyclists were quite different from their corresponding metagenomic profiles. *Prevotella* was less abundant in the metatranscriptome yet those cyclists in cluster one (Fig. 1a) still grouped together. And while there were fewer significant correlations between *Prevotella* and KEGG pathways with RNA-Seq read data, there was still strong agreement in what type of metabolic pathways were enriched for when *Prevotella* was more abundant with regards to both mWGS and RNA-Seq read data. Of interest was the upregulation of branched chain amino acid (BCAA) biosynthesis when there was an increase in *Prevotella* transcripts and the corresponding downregulation in the degradation of BCAAs from the mWGS dataset when *Prevotella* was highly abundant. High levels of BCAAs (leucine, isoleucine, and valine) are known to decrease exercise-induced muscle fatigue and promote muscle-protein synthesis [36]. While there is strong evidence showing that BCAAs do not enhance exercise performance [36, 37], they are shown to reduce central fatigue through several pathways and attenuate muscle damage during prolonged endurance exercise [38]. Since BCAAs are not produced by the human body and need to come from the diet, having a gut community that contains *Prevotella* spp. to either synthesize BCAAs or alternatively influence other microbes to produce these amino acids would be highly beneficial to athletes that need fast recovery time from intense exercise.

Similar to *Prevotella*, *M. smithii* is positively associated with diets high in carbohydrates [22]. *M. smithii* increases the fermentation efficiency of many bacterial taxa in the gut, including those that ferment complex polysaccharides, through its utilization of hydrogen gas (H_2_) and formate to reduce carbon dioxide (CO_2_) to methane [39, 40]. Without the presence of a methanogen such as *M. smithii* to utilize these fermentation productions, the accumulation of H_2_ in the distal colon directly inhibits bacterial NADH dehydrogenases, thereby decreasing production of ATP, SCFAs, and other important compounds [39, 40]. Therefore, the unique metabolism of *M. smithii* allows the microbial community to be more energetically efficient and this effect has been demonstrated in a human gnotobiotic mouse model [41]. This would be of direct benefit for athletes because an increase in degradation of organic matter in the distal colon results in an increase in bacterial fermentation products (such as SCFAs) that would be absorbed and utilized by the host. Theoretically, this could lead to reduced recovery time from intense exercise and may even influence race performance. Indeed the “blooms” of *M. smithii* activity from the cyclists and the subsequent correlations between methane metabolism and pathways involved in carbohydrate and energy metabolism suggest a more energetically efficient gut microbiome when *M. smithii* is active. What would directly trigger such a bloom of activity is of interest for further investigation. While it is possible that there could have been some bias towards enrichment of *M. smithii* transcripts due to increased stability of *M. smithii* mRNA compared to bacterial mRNA, we do not feel this bias negatively influenced results. Necessary precautions were taken in the collection and handling of all samples. We observed varying amounts of mRNA reads from *M. smithii* across all samples, from a small portion of total mRNA (<1%) to 41% of all transcripts*.* In addition, the types of transcripts enriched for across cyclists’ who had increased abundance of *M. smithii* all showed similar trends in what pathways were most expressed, most notably methanogenesis.

In addition to understanding what would drive a bloom of *M. smithii*, there is interest in determining why there is such a high percentage of colonization by *M. smithii* in professional cyclists. Our results raise several questions: would a given cyclist have an increased chance of becoming a professional athlete if they were colonized by *M. smithii* due to increased metabolism of the gut microbiome? Or would a lifetime of training, competition, and a diet high in complex carbohydrates somehow influence a better niche for *M. smithii* to thrive where the gut is constantly subjected to physiological perturbations? Exhausting endurance events are known to increase colonic transit time [42] which would allow for increased growth time for archaea such as *M. smithii* [39]*.* And despite some studies that correlate the presence of *M. smithii* with intestinal-related disorders including irritable bowel syndrome (IBS) and constipation [43, 44], no cyclists reported IBS or constipation-related issues during the consent process. Future studies should aid in our understanding of why *Methanobrevibacter smithii* is more common in professional cyclists. It will also be interesting to see if this archaeon is more common in other types of professional athletes compared to amateurs and/or non-athletes.

## Conclusions

This pilot study provides the first look into the gut microbiomes of cyclists, and we have found significant correlations between what taxa are present in professional cyclists and what taxa correlate to high exercise load. These data present opportunities for generating important hypotheses regarding how intense training influences the microbiome in cyclists. It is likely that multiple factors influence how the gut communities of athletes are structured including the type of exercise, amount of exercise, diet, host immunity, host metabolism, and the physiological aspects of the human gut including bile acid secretion and transit time. Further studies will be important for understanding the impact of these factors on the metabolic capacity of the gut microbiome and how organisms such as *Prevotella* and *Methanobrevibacter* may respond to exercise and, in turn, positively influence health and athletic performance.

## Methods

### Sample collection and nucleic acid extraction

Thirty-three cyclists (11 females, 22 males), aged 19–49 (median age 33), with no major medical issues and no antibiotic use within the previous year were enrolled to provide fecal samples. The organization that governs competitive cycling events, USA cycling (https://www.usacycling.org), designates four racing classes overall: professional, category 1 (CAT 1), category 2 (CAT 2), and category 3 (CAT 3). These four classes are split based on overall fitness, skill level, and time needed to complete a certain race track. Professional level racers are the fastest on a given track. CAT 1 level racers are one class below professional, followed in lessening skill and race time by CAT 2 and CAT 3. CAT 3 racers are the slowest (i.e., entry level) and overall least fit. We recruited racers from professional level (the highest level) and CAT 1 level (amateur) racers. We refrained from collecting samples from entry-level racers (CAT 3 and CAT 2 levels) because the goal was to look at highly fit individuals that had been competing for ≥2 years. Twenty-two cyclists in our cohort were classified by their USA cycling licenses as professional level racers and 11 cyclists were classified as CAT 1 (amateur) racers (Table 1). Cyclists filled out questionnaires on diet, alcohol consumption, and the average number of hours of exercise per week. All participants spent a minimum of 6 h exercising per week.

Fecal samples were self-collected in polyethylene sample collection containers (Fisher Scientific) with a portion (approx. 2–5 g) placed in a 50 mL conical tube containing 20 mL RNALater (Qiagen). Samples were immediately placed on frozen freezer packs, were shipped overnight to the Jackson Laboratory for Genomic Medicine, and were immediately stored at −80 °C. DNA from stool was extracted using the PowerSoil DNA Isolation Kit (MO BIO Laboratories, Inc.). RNA was extracted from stool using the PowerMicrobiome RNA Isolation Kit (MO BIO Laboratories, Inc.) followed immediately by an additional DNAse treatment using the Turbo DNA-Free Kit (Life Technologies). All nucleic acids were quantified using the Qubit® assay (Life Technologies) and stored at −80 °C. The integrity of all RNA samples was assessed using the Agilent RNA 6000 Nano Kit (Agilent Technologies).

### mWGS library preparation and sequencing

mWGS libraries were generated using the TruSeq Nano DNA Sample Preparation Kit (Illumina). Samples were pooled at equal nanomolar concentration and either 125- or 150-base paired-end reads were generated on the Illumina NextSeq and HiSeq instruments. Duplicated sequences were removed and human contaminant sequences were filtered out using BMTagger [45]. Adapters and low quality bases were trimmed using Flexbar [46] and low-complexity sequences were masked using Dustmasker [47]. The resulting reads were termed ‘clean’ and were used for further taxonomic and metabolic function analysis.

### Metatranscriptomic (RNA-Seq) library preparation and sequencing

Total RNA was isolated, ribosomal RNA was removed, and mRNA libraries were generated with the ScriptSeq V2 RNA-Seq Complete Gold Kit for Epidemiology (Epicenter). cDNA samples were pooled at equal nanomolar concentration and 150-base paired-end reads were generated on the Illumina NextSeq instrument. Sequences from each sample were trimmed of adapters using Trimmomatic [48]. Primer, rRNA, tRNA, phiX, and human contaminant sequences were removed using the Burrows-Wheeler Aligner (BWA) [49]. The resulting reads were termed clean and were used for further taxonomic and metabolic function analysis.

### Bioinformatic analysis of mWGS and RNA-Seq data

For taxonomic assignment, all cleaned mWGS and RNA-Seq reads were aligned to the Real Time Genomics™ (RTG) database v2.0 [50] utilizing RTG’s “map” and “species” modules and the BWA [49]. The top 25 most abundant genera in each sample were calculated in R (v3.1) [51]. The Bray-Curtis (BC) dissimilarity index and the average-linkage method were used for clustering and dendrograms were created using the Interactive Tree of Life (ITOL) software [52, 53]. Approximately, unbiased *p* values were calculated using the R package “pvclust”. For functional assignment of mWGS and RNA-Seq data, open reading frames were predicted on assembled, cleaned sequences using FragGeneScan [54] v1.19. Protein coding sequences were clustered at 90% identity using “cluster_fast” from USEARCH v8.0 [55]. The amino acid sequence representing each cluster, as well as all unclustered sequences, were aligned to the KEGG database using BLASTP (BLAST + 2.2) [47]. The *E* value cutoff for KEGG annotation was <0.01. Fisher’s exact test, the Wilcoxon rank-sum test, and Spearman’s rank correlation coefficients with corresponding *p* values were calculated in R.

For insight into what genes were most highly expressed by *Methanobrevibacter smithii* in the cyclists, RNA-Seq reads from eight professional cyclists with high activity of *M. smithii* (>8% transcript relative abundance) were aligned to the reference genome *Methanobrevibacter smithii* ATCC 35061 using the BWA. Potential PCR duplicates were removed with SAMtools [56]. SAMtools and BEDtools [57] were used to determine the number of reads hitting each CDS, and the output was normalized using reads per kilobase mapped (RPKM). For comparison of gene expression profiles between samples, RPKM values were converted to transcripts per million (TPM) by RSEM software [58]. Heatmaps were produced in R, and hierarchical clustering was determined using the BC dissimilarity index and average-linkage method (Additional file 15).

## Additional files

Additional file 1: Table showing the average read depths for mWGS sequencing, 16S rRNA gene sequencing, and RNA-Seq. (XLSX 30 kb)

Additional file 2: Table showing mWGS taxonomic abundance data for all genera. (XLSX 151 kb)

Additional file 3: Figure showing taxonomic clustering with 16S rRNA gene sequencing at the genus level. (TIFF 439 kb)

Additional file 4: Table showing mWGS read-based KEGG pathway abundance table. (XLSX 86 kb)

Additional file 5: Table showing mWGS taxonomic abundance data for all species. (XLSX 349 kb)

Additional file 6: Figure characterizing the *Prevotella* and *Bacteroides* species in cyclists. (TIFF 4233 kb)

Additional file 7: Table showing all 16S rRNA sequencing-based OTUs belonging to *Prevotella.* (XLSX 48 kb)

Additional file 8: Table showing mWGS taxonomic abundance data for all phyla. (XLSX 55 kb)

Additional file 9: Table showing RNA-Seq taxonomic abundance data for all phyla. (XLSX 49 kb)

Additional file 10: Figure illustrating phylum-level differences between the metagenome and metatranscriptome. (TIFF 201 kb)

Additional file 11: Table showing RNA-Seq taxonomic abundance data for genera. (XLSX 145 kb)

Additional file 12: Table showing RNA-Seq taxonomic abundance data for species. (XLSX 323 kb)

Additional file 13: Table showing abundance of *M. smithii* based on mWGS sequencing, RNA-Seq, and qPCR. (DOCX 99 kb)

Additional file 14: Table showing RNA-Seq read-based KEGG pathway abundance data. (XLSX 110 kb)

Additional file 15: Materials and methods used for additional files. (DOCX 122 kb)
