# Supplementary figures and images for: Community characteristics of the gut microbiomes of competitive cyclists
Source: Microbiome. 2017 Aug 10;5:98. doi: 10.1186/s40168-017-0320-4 (PMC5553673; doi:10.1186/s40168-017-0320-4)

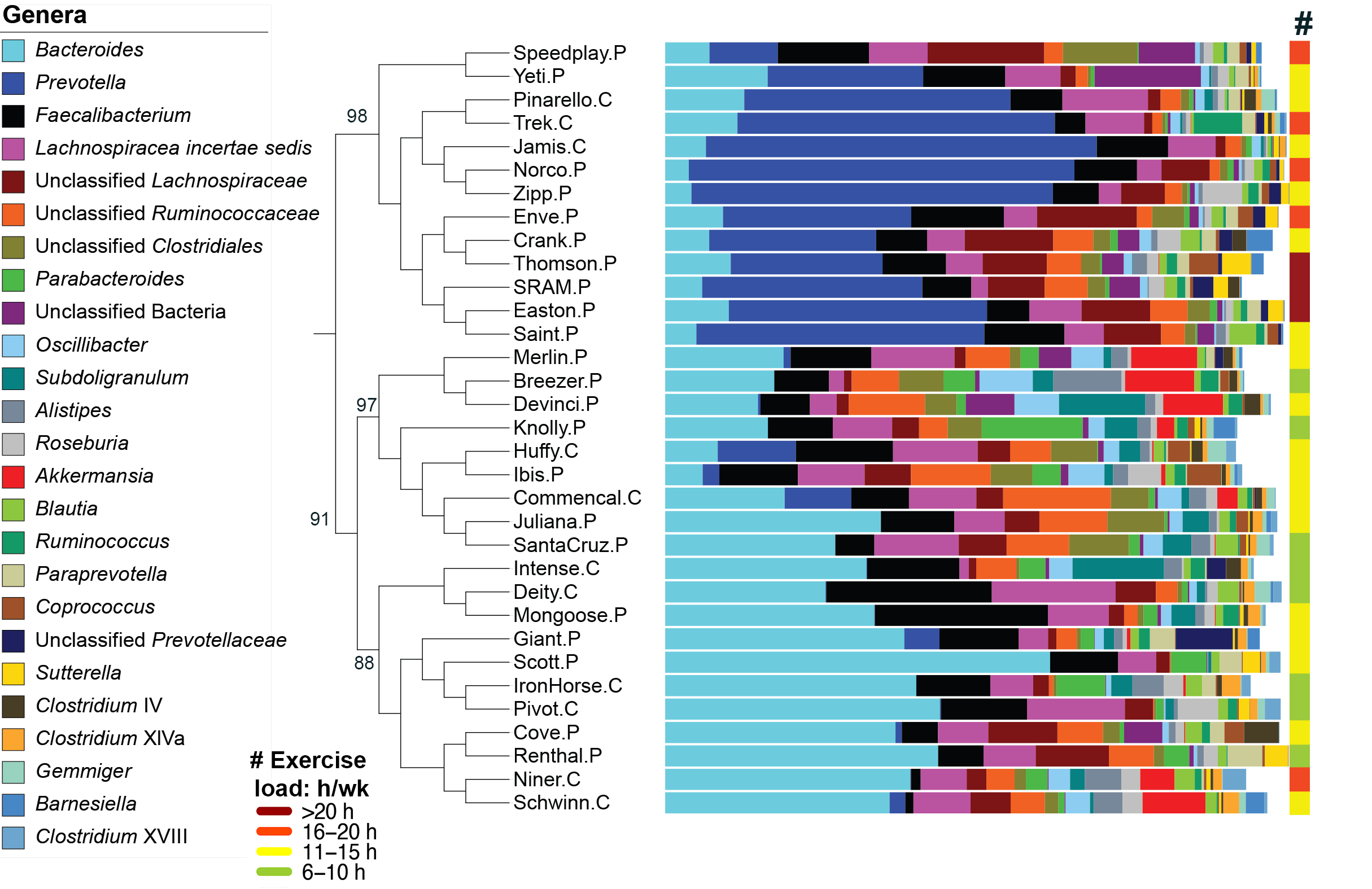

Supplement: Supplementary file 3 — Figure showing taxonomic clustering with 16S rRNA gene sequencing at the genus level. (TIFF 439 kb) [file 40168_2017_320_MOESM3_ESM.tif]

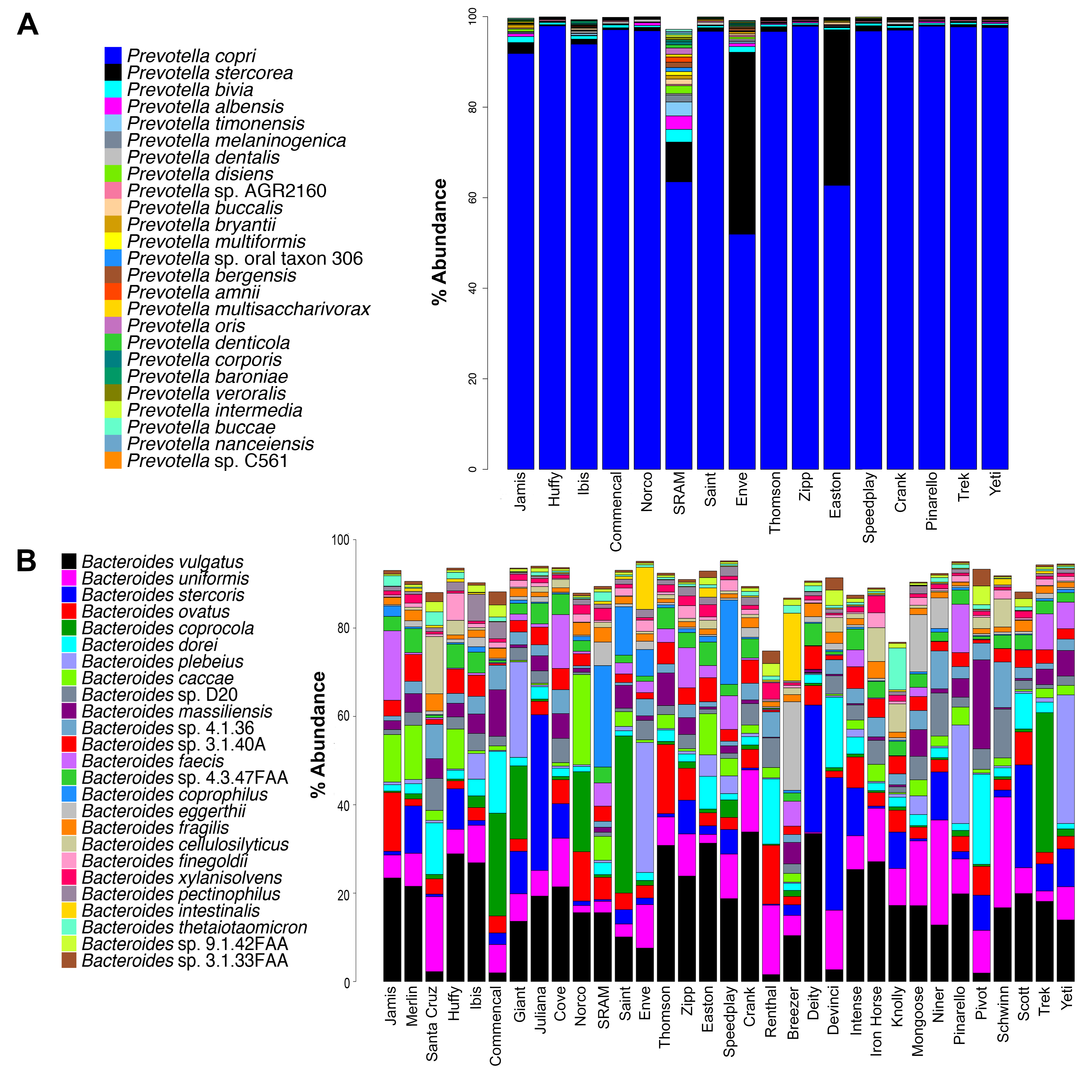

Supplement: Supplementary file 6 — Figure characterizing the Prevotella and Bacteroides species in cyclists. (TIFF 4233 kb) [file 40168_2017_320_MOESM6_ESM.tif]

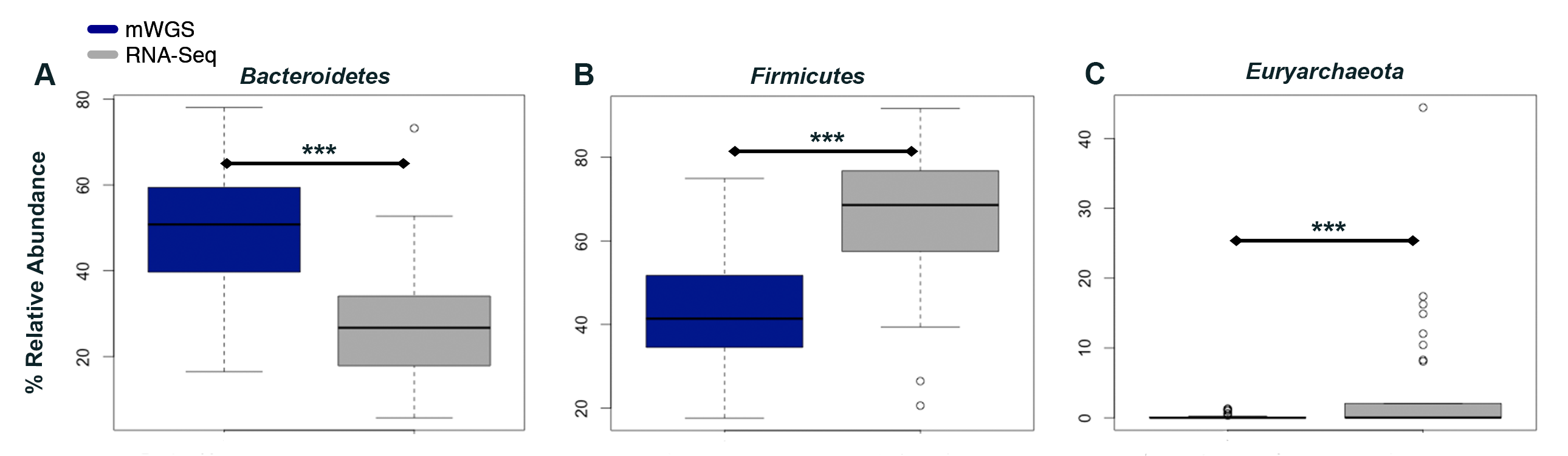

Supplement: Supplementary file 10 — Figure illustrating phylum-level differences between the metagenome and metatranscriptome. (TIFF 201 kb) [file 40168_2017_320_MOESM10_ESM.tif]
