# Supplementary material for: Community characteristics of the gut microbiomes of competitive cyclists
Source: Microbiome. 2017 Aug 10;5:98. doi: 10.1186/s40168-017-0320-4 (PMC5553673; doi:10.1186/s40168-017-0320-4)
Supplement: Supplementary file 13 — Table showing abundance of M. smithii based on mWGS sequencing, RNA-Seq, and qPCR. (DOCX 99 kb) [file 40168_2017_320_MOESM13_ESM.docx]

| **Racer** | **mWGS Taxonomic Cluster^A^** | **Percent Abundance *M.smithii:* RNA-Seq^B^** | **Percent Abundance *M.smithii:* mWGS^C^** | **Average qPCR**  **Ct Value^DE^** |
| --- | --- | --- | --- | --- |
| **Pro Racers:** |  |  |  |  |
| Breezer | Two | **41.0%** | 0.4% | 25.57 ± 0.11 |
| Knolly | Three | **16.2%** | 0.7% | 22.99 ± 0.31 |
| Merlin | Three | **12.8%** | 0.4% | 25.51 ± 0.39 |
| Devinci | Three | **11.0%** | **1.0%** | 23.35 ± 0.10 |
| Ibis | Three | **10.4%** | **1.5%** | 21.49 ± 0.28 |
| Giant | Two | **9.5%** | 0.3% | 25.01 ± 0.37 |
| SpeedPlay | Three | **8.3%** | 0.0% | 36.88 ± 1.36 |
| SRAM | Three | **8.1%** | 0.6% | 22.96 ± 0.93 |
| Enve | Three | **2.1%** | 0.1% | 27.02 ± 0.16 |
| Juliana | Two | 0.5% | 0.1% | 28.81 ± 0.17 |
| Saint | One | 0.05% | 0.0% | 35.91 ± 0.65 |
| Crank | Three | 0.05% | **1.1%** | 24.99 ± 0.14 |
| Norco | One | 0.04% | 0.008% | 30.97 ± 0.22 |
| Mongoose | Three | 0.3% | 0.1% | 30.52 ± 0.20 |
| Yeti | Three | 0.2% | 0.0% | Undetermined |
| Scott | Two | 0.0% | 0.0% | Undetermined |
| Santa Cruz | Two | 0.0% | 0.0% | Undetermined |
| Renthal | Two | 0.0% | 0.0% | Undetermined |
| Zipp | One | 0.0% | 0.0% | Undetermined |
| Cove | Two | 0.0% | 0.0% | Undetermined |
| Easton | One | 0.0% | 0.0% | Undetermined |
| Thomson | Three | 0.0% | 0.0% | Undetermined |
| **Amateur (CAT 1) Racers:** |  |  |  |  |
| Trek | One | **1.2%** | 0.04% | 26.39 ± 0.02 |
| Pivot | Two | 0.0% | 0.0% | 32.91 ± 0.50 |
| Iron Horse | Two | 0.0% | 0.0% | Undetermined |
| Jamis | One | 0.0% | 0.0% | Undetermined |
| Commencal | Three | 0.0% | 0.0% | Undetermined |
| Pinarello | One | 0.0% | 0.0% | Undetermined |
| Deity | Three | 0.0% | 0.0% | Undetermined |
| Intense | Two | 0.0% | 0.0% | Undetermined |
| Niner | Two | 0.0% | 0.0% | Undetermined |
| Huffy | Three | 0.0% | 0.0% | Undetermined |
| Schwinn | Two | 0.0% | 0.0% | Undetermined |

^A^ Cluster was determined based on mWGS sequencing data.

^BC^ Bold text indicates the % abundance is > 1% from the metagenome^B^ or metatranscriptome^C^.

^D^ Genomic DNA samples were tested by qPCR for presence of *M. smithii* (in triplicate) using primers specific for the *M. smithii* 16S rRNA gene.

^E^ Undetermined indicates the primers failed to amplify and therefore no *M. smithii* was detected in that sample.
