# Supplementary material for: Community characteristics of the gut microbiomes of competitive cyclists
Source: Microbiome. 2017 Aug 10;5:98. doi: 10.1186/s40168-017-0320-4 (PMC5553673; doi:10.1186/s40168-017-0320-4)
Supplement: Supplementary file 15 — Materials and methods used for additional files. (DOCX 122 kb) [file 40168_2017_320_MOESM15_ESM.docx]

**Additional Materials and Methods**

**Metagenomic 16S rRNA gene sequencing and data processing**

The V1-V3 region of the 16S rRNA gene was amplified with modified primers 27F (5’-AATGATACGGCGACCACCGAGATCTACACNNNNNNNNA CACTCTTTCCCTACACGACGCTCTTCCGATCTAGAGTTTGATCCTGGCTCAG-3’) and 534R (5’-CAAGCAGAAGACGGCATACGA GATNNNNNNNNGTGACTGGAGTTCAGACGTGTGCTCTTCCGATCTATTACCGCGGCTGCTGG-3’) where the Ns represent a unique 8 bp index, the 5’ end contains Illumina platform adapter sequences, and the 3’ end contains the primer sequence for the 16S rRNA gene. PCR mixtures contained 1 μM of each forward and reverse primer, 4 ng template DNA, 0.75 U AccuPrime *Taq* High Fidelity DNA Polymerase (Life Technologies), and 2 μl AccuPrime Buffer II (Life Technologies) in a final volume of 20 μl. Thermal cycling consisted of an initial denaturation step at 95°C for 2 min, followed by 30 cycles of denaturation at 95°C for 20 s, annealing at 56°C for 30 s and extension at 72 °C for 60 s, with a final extension step at 72°C for 5 min. PCR products were purified using 1.8X volume Agencourt AMPure XP beads (Beckman Coulter) and quantified using the Qubit® dsDNA BR assay or dsDNA HS assay (Life Technologies).

For sequencing, samples were pooled at equal nanomolar concentration and 300 base paired end reads were generated on the Illumina MiSeq instrument (Illumina, San Diego, CA, USA). Sequences were trimmed of primer sequences using Trimmomatic [1], paired ends were assembled using FLASH [2] (10% maximum mismatches allowed), and chimeras were removed using UCHIME [3]. All cleaned, assembled sequences were aligned to the RDP database [4] v2.10.1 using RDP-classifier with a 50% confidence value threshold. The top 25 most abundant genera in each sample were calculated in R (v3.1) [5]. The Bray-Curtis (BC) dissimilarity index and the average-linkage method were used for clustering and dendrograms were created using the Interactive Tree of Life (ITOL) software [6,7]. Approximately unbiased *p*-values were calculated using the R package “pvclust”.

To identify the operational taxonomic units (OTUs) belonging to the genus *Prevotella* in cyclists based on 16S gene data, sequencing reads were grouped into operational taxonomic units (OTUs) using the centroid-based clustering as implemented in the UPARSE-OTU algorithm [8]. Sequences with distance-based similarity of 97% or greater were assigned to the same OTU. OTU sequences were aligned to the NCBI 16S ribosomal RNA sequence database using megablast with an E-value cutoff of <0.01. The hit with lowest E-value and highest percent identity was used for taxonomic assignment.

**Quantitative PCR**

Detection and relative quantification of *Methanobrevibacter smithii* was done on a ViiA7 qPCR machine (Life Technologies) using TaqMan Fast Advanced Master Mix (Applied Biosystems), 0.3 μM primers, 0.3 μM probe, and a 1:10,000 dilution of DNA (20-150 ng/μL stock). The 16S rDNA primers specific for *M. smithii* were designed previously [9]. The PCR amplification program was 95°C for 2 min, following by 40 cycles of 95°C for 1 sec and 60°C for 45 sec. Approximately 0.05 ng of DNA was used per reaction and all samples were tested in triplicate.

**References**

1. Bolger AM, Lohse M, Usadel B. Trimmomatic: a flexible trimmer for Illumina sequence data. Bioinformatics. 2014, 170:1–7.

2. Magoč T, Salzberg SL. FLASH: Fast length adjustment of short reads to improve genome assemblies. Bioinformatics. 2011, 27:2957–63.

3. Edgar RC, Haas BJ, Clemente JC, Quince C, Knight R. UCHIME improves sensitivity and speed of chimera detection. Bioinformatics. 2011, 27:2194–200.

4. Wang Q, Garrity GM, Tiedje JM, Cole JR. Naïve Bayesian classifier for rapid assignment of rRNA sequences into the new bacterial taxonomy. Appl. Environ. Microbiol. 2007, 73:5261–7.

5. Hornik K. The comprehensive R archive network. Comput. Stat. 2012, 4:394–8.

6. Letunic I, Bork P. Interactive Tree Of Life (iTOL): An online tool for phylogenetic tree display and annotation. Bioinformatics. 2007, 23:127–8.

7. Letunic I, Bork P. Interactive Tree of Life v2: Online annotation and display of phylogenetic trees made easy. Nucleic Acids Res. 2011, 39:475–8.

8. Edgar RC. UPARSE: Highly accurate OTU sequences from microbial amplicon reads. Nat. Methods. 2013, 10:996–8.

9. Dridi B, Henry M, El Khéchine A, Raoult D, Drancourt M. High prevalence of Methanobrevibacter smithii and Methanosphaera stadtmanae detected in the human gut using an improved DNA detection protocol. PLoS One. 2009, 4:e7063.
